# AGE-Rich Bread Crust Extract Boosts Oxidative Stress Interception via Stimulation of the NRF2 Pathway

**DOI:** 10.3390/nu13113874

**Published:** 2021-10-29

**Authors:** Kristin Wächter, Alexander Navarrete Santos, Anne Großkopf, Tim Baldensperger, Marcus A. Glomb, Gábor Szabó, Andreas Simm

**Affiliations:** 1 Department for Cardiac Surgery, University Hospital Halle (Saale), Martin-Luther University Halle-Wittenberg, 06120 Halle (Saale), Germany; anne.grosskopf@uk-halle.de (A.G.); gabor.szabo@uk-halle.de (G.S.); andreas.simm@uk-halle.de (A.S.); 2 Center for Medical Basic Research, Martin-Luther-University Halle-Wittenberg, 06120 Halle (Saale), Germany; alexander.navarrete@uk-halle.de; 3 German Institute of Human Nutrition Potsdam-Rehbrücke, 14558 Nuthetal, Germany; tim.baldensperger@dife.de; 4 Institute of Chemistry, Food Chemistry, Martin-Luther-University Halle-Wittenberg, 06120 Halle (Saale), Germany; marcus.glomb@chemie.uni-halle.de

**Keywords:** advanced glycation end products, bread crust extract, NRF2, oxidative stress resistance, functional food, cardioprotection

## Abstract

Advanced glycation end products (AGEs) result from a non-enzymatic reaction of proteins with reactive carbohydrates. Heat-processed food, such as bread, contains high amounts of AGEs. The activation of the NF-κB signaling pathway by bread crust extract (BCE) is well understood. However, it is largely unknown whether NRF2, the master regulator of oxidative stress resistance in mammalian cells, is affected by BCE. We have investigated the molecular mechanisms by which BCE induces antioxidant gene expression in cellular models. Our data showed that soluble extracts from bread crust are capable of stimulating the NRF2 signaling pathway. Furthermore, NRF2 pathway activation was confirmed by microarray and reporter-cell analyses. QRT-PCR measurements and Western blot analyses indicated an induction of antioxidative genes such as HMOX1, GCLM and NQO1 upon BCE treatment. Moreover, BCE pretreated cells had a survival advantage compared to control cells when exposed to oxidative stress. BCE induces phosphorylation of AKT and ERK kinase in EA.hy926 cells. By mass spectrometry, several new, potentially active modifications in BCE were identified. Our findings indicate that BCE activates NRF2-dependent antioxidant gene expression, thus provoking a protection mechanism against oxidative stress-mediated tissue injury. Hence, BCE can be considered as functional food with antioxidative and cardioprotective potential.

## 1. Introduction

Advanced glycation end products (AGEs) or Maillard reaction products (MRPs) are generated by non-enzymatic reactions of protein amino groups with reducing sugars [1]. Heat treatment of food, such as cooking or baking, induces the development of AGEs by the browning reaction, i.e., the Maillard reaction. Bread crust is an example of a food product rich in AGEs. Typical AGEs identified in bread crust are pyrraline, carboxymethyl-lysine (CML) and methylglyoxal hydroimidazolone [2,3]. Beside AGE-modified proteins present in bread crust, “pronylated” proteins were proposed to mediate antioxidant and chemopreventive activity [4]. Further positive effects were observed on the enzymatic defense of the liver such as increased expression of catalase and glutathione peroxidase after feeding rats with bread crust [5]. An increased antioxidant state in the plasma of bread crust fed rats and the up-regulation of chemopreventive enzymes such as NADPH- Cytochrome- C- Reductase and Glutathione-S-Transferase were assessed by Somoza and colleagues [6]. In mouse cardiac fibroblasts stimulated with extracts from bread crust, intracellular oxidative stress was induced [2]. This led to an increased manganese superoxide dismutase (MNSOD) expression and prevention against H_2_O_2_ induced cell death [7]. Bartling and colleagues observed non-small cell lung carcinoma growth in mice fed with bread crust vs. a control group and found that mice with higher AGE serum levels developed smaller tumors [8]. It is well known that MRPs rich foods such as bread crust induce the NF-κB signaling pathway [2,9]. An additional, potentially influenced transcription factor is the nuclear factor E2-related factor 2 (NRF2), since MRPs and coffee activate NRF2 in macrophages and Caco-2 cells [10] and AGE-modified bovine albumin (AGE-BSA) induces NRF2 in endothelial cells [11]. NRF2 is an evolutionarily conserved master regulator of detoxification, antioxidant, anti-inflammatory and cytoprotective mechanisms (reviewed in [12,13]). Under physiological conditions, NRF2 is bound and inhibited by KEAP1. Upon oxidative or electrophilic stress, the NRF2-KEAP1 complex is disrupted and NRF2 can enter the nucleus [14]. Binding of NRF2 to an antioxidant response element (ARE) leads to activation of genes that encode detoxifying and antioxidant enzymes such as HMOX1, NQO1 and GCLM [15,16,17]. NRF2 is also involved in the modulation of ferroptosis, a regulated cell death dependent on iron and ROS accumulation [18], first reported by Dixon and co-workers in 2012 [19].

As AGEs from food will enter the blood stream and can act primarily on endothelial cells, we studied the effect of bread crust on signaling pathways, antioxidative defense mechanisms and global gene expression in endothelial cells. Furthermore, NRF2–EA.hy926-reporter-cell analyses uncovered that bread crust extract (BCE) stimulates the NRF2 pathway.

## 2. Materials and Methods

### 2.1. Western Blot Analysis

For Western blotting, cytoplasmic protein extracts were prepared with Tris–NP-40 buffer (25 mM Tris, 150 mM NaCl, 2 mM EDTA, 1% NP-40, 1x protease inhibitor (Sigma-Aldrich, MO, USA), pH 7.6). For analyses of phosphorylated proteins, 5 mM sodium orthovanadate was added to the lysis buffer. Denatured protein lysates were separated by SDS-PAGE and transferred on nitrocellulose membranes (GE Healthcare Life Sciences, MA, USA) by tank blotting (Bio-Rad, Germany) using a blotting buffer (25 mM Tris, 150 mM Glycine, 10% Methanol (*v*/*v*)). Afterwards, membranes were blocked (5% milk) and incubated with primary and appropriate secondary antibodies (please refer to Table A1) and analyzed by infrared scanning using a LI-COR Odyssey scanner (LI-COR Biosciences, Lincoln, NE, USA).

### 2.2. RNA Extraction, Reverse-Transcription, and quantitative Real-Time Polymerase Chain Reaction (qRT-PCR)

Total RNA was isolated from cells by TRIzol extraction. For this purpose, cells were washed with 1xPBS solution (Thermo Fisher Scientific, MA, USA; 10010-015) and harvested with TRIzol Reagent (Thermo Fisher Scientific, MA, USA; 15596018; 1 mL per 6-well). After adding chloroform (Sigma Aldrich, MO, USA; 32211-1L-M; 200 µL), mixing and centrifugation (2000*g, 5 min) the upper phase was gently agitated with isopropanol (Sigma Aldrich; MO, USA; 33539-1L-M; 1:1 *v*/*v*) and incubated for one hour at room temperature. RNA was pelletized by centrifugation (14,000× *g*, 10 min, 4 °C) and the pellet was washed three times with 80% ethanol. After pellet drying, nuclease free water was added, and the RNA was stored at −20 °C. According to the protocol of iScript Advanced cDNA Synthesis Kit for RT-qPCR (Bio-Rad, Germany) RNA (3 µg) was incubated with 5× iScript Advanced Reaction Mix together with iScript Advanced Reverse Transcriptase (total 20 μL) at 46 °C for 20 min and at 95 °C for 1 min. The obtained complementary DNAs (cDNAs) were used for quantitative real-time polymerase chain reaction (qRT-PCR) using SsoAdvanced Universal SYBR Green Supermix (Bio-Rad, Germany) together with specific forward and reverse primers (listed in Table A2). The amplification was performed on the CFX Connect Real-Time System (Bio-Rad, Germany) under the following conditions: at 95 °C for 3 min, 45 cycles at 95 °C for 30 s, at 60 °C for 30 s, and at 72 °C for 30 s. The mRNA expression levels of the target genes were normalized by the levels of internal reference genes (Beta actin and Y-box binding protein 1) and calculated according to the DDCt method. Data were analyzed from three independent experiments.

### 2.3. Microarray Analyses

For array analyses, an additional RNA purification step was included by using RNeasy MinElute Cleanup Kit (Quiagen, Netherlands; 74204) according to the manufacturer instructions. To ensure quality and integrity for subsequent array analyses, RNA was dissected by Bioanalyzer (2100 Bioanalyzer, Agilent, CA, USA; supplemental Figure S1A,B). Detection of RNA was performed by microarray (Clariom™ D Assay, Thermo Fisher Scientific, MA, USA). Biotin-labeled ss-cDNA was synthesized from total RNA with an GeneChip^TM^ WT PLUS Reagent Kit (Thermo Fisher Scientific, MA, USA) with a subsequent GeneChip hybridization procedure using Clariom D human arrays (Thermo Fisher Scientific, MA, USA) and GeneChip Fluidics station 450 (Thermo Fisher Scientific, MA, USA). Hybridized mRNA chips were washed and were scanned by the Affymetrix GeneChip Scanner 7G with GeneChip Command Console 3.1 software. Data calculation was performed with the Transcriptome Analysis console (TAC 4.0; applied biosystems; Thermo Fisher Scientific, MA, USA). Differentially expressed genes were identified through fold change (up-regulated > 2.0; down-regulated < 2.0) as well as *p*-value < 0.01. Functional annotation of the gene list was conducted with the consensus path DB human (cpdb.molgen.mpg.de; release 34; [20]) for identification of enriched pathways. To understand the interplay of regulated RNAs, network analyses were performed (https://string-db.org; STRING 11.0b; 26 November 2020 [21]). Hierarchical clustering helped to display expression patterns among the samples (TAC 4.0; applied biosystems; Thermo Fisher Scientific, MA, USA).

### 2.4. Cell Culture, Transfection, Inducers and Inhibitor

EA.hy926 and HeLa cells (obtained from ATCC^®^ CRL-2922™, somatic cell hybrid established by fusing primary HUVEC with a thioguanine-resistant clone of A549; ATCC^®^ CCL-2, cervical cancer cells; passage No. 15-20; VA, USA) were cultured in Dulbecco’s Modified Eagle Medium; 4.5 g/L Glucose (DMEM; Thermo Fisher Scientific, MA, USA) containing 10% FCS (0.1% FCS under starvation) and 100 U/mL Penicillin and 100 µg/mL Streptomycin in a 37 °C humidified incubator in the presence of 10% CO_2_. EA.hy926 reporter cells (NRF2-ARE-mCherry fluorescence based, see Figure 4a) were generated by transfection of plasmid (CS-13227-LvGN01; GeneCopoeia, MD, USA) by FuGene HD Transfection reagent (Promega, WI, USA). Transfection efficiency was improved by transfection of linearized and shortened plasmid (NsbI-AjuI-digest; NEB, MA, USA). Positive cells were selected by puromycin treatment (0.25 µg/mL) and sorted by flow cytometry (BD FACS Aria2; NJ, USA; more than 4-fold over background) after stimulation with 20 ng/mL Phorbol 12-myristate 13-acetate (Sigma-Aldrich, MO, USA). On 6-well dishes, 3 × 10^5^ cells per well were seeded and, where indicated, cells were incubated with different NRF2-inducers (5 µM, 10 µM sulforaphane; LKT Laboratories, MN, USA; and 4 µM, 8 µM falcarinol; Cayman chemical, MI, USA) or inhibitors (10 nM, 30 nM, 100 nM, 300 nM brusatol; Carbosynth, UK).

### 2.5. Hydrogen Peroxide Stress Assay

Five hours after seeding, cells were incubated with 3 µg/mL BCE for 18 h. Medium was changed and five hours later 3 × 10^4^ cells were seeded per well in 12-well plates. The next day H_2_O_2_ (0.25 mM, 0.5 mM and 0.75 mM; University pharmacy Halle) was applied for 4 h, then the medium was changed. Twenty hours later, cells were harvested and counted by using a CASY (Schärfe System, Germany) device. The CASY system counts cells by using electronic current exclusion (ECE) and pulse field analysis allowing the determination of cell viability. The membrane of viable cells offers a barrier for the current; therefore, the full cell volume is measured, whereas in case of dead cells only the nucleus is detected.

### 2.6. Statistical Analysis

The data were presented as mean values with standard deviation, n represents the number of independent experiments. For statistical analysis of data, the 2-tailed, paired Student’s T test was calculated. Results with *p* < 0.05 were considered statistically significant (* *p* < 0.05; ** *p* < 0.01; *** *p* < 0.001).

### 2.7. Production of Bread Crust (BC) and Preparation of Bread Crust Extract (BCE)

Bread was baked as a mixture of rye flour, wheat flour, yeast, sourdough and sodium chloride (NaCl) as previously described [4]. The chloroform defatted and lyophilized bread crust powder was stored at −20 °C. A soluble bread crust extract (BCE; 250 mg bread crust powder in 1 mL PBS (GIBCO, Karlsruhe, Germany)) was prepared by a 3 min. sonication followed by a centrifugation step at 867× *g* for 30 min at 10 °C and a second centrifugation step at 14,000× *g* for 30 min at 4 °C. The supernatant was sterile filtered (0.1 µm PES membrane filter; VWR, PA, USA) and stored at −20 °C until further use.

### 2.8. TCA-Precipitation, Acidic Hydrolysis and Mass Spectrometric Identification of Advanced Glycation End Products in BCE

BCE was subjected to total acidic hydrolysis to determine the AGE-content within existing protein fragments. First, 3 mg aliquots of BCE were mixed with an equal volume of 20% trichloracetic acid (TCA) and incubated on ice for 1 h. Precipitated protein fragments were pelletized at 14,000× *g* for 15 min at 4 °C and washed four times with 5% of TCA, each time followed by another centrifugation step at the previously described settings. Subsequently, samples were evaporated to dryness in an Eppendorf concentrator 5301 at room temperature. Next, the dried BCE-precipitate was resolved in PBS, transferred to a WHEATON glass reaction tube and subjected to reduction and acidic hydrolysis as described before [22] with slight changes. Shortly, reduction was carried out by the addition of LiBD_4_ for 1 h, at RT. The reaction was stopped by the addition of 1 M HCl and neutralized by 1 M NaOH. Aliquots were then dried and hydrolyzed in 6 N HCl for 20 h at 110 °C under a nitrogen atmosphere. After vacuum concentration, the aliquots were resolved in ultra-pure water, filtered through a 0.45 µm cellulose acetate spin filter (Costar SpinX, Corning Inc., USA) and subjected to identification of AGEs and other putatively active modifications via mass spectrometry. An analytical HPLC-MS/MS setup comprised of a PU-2080 plus quaternary gradient pump with degasser, a AS-2057 plus autosampler (Jasco, Gross-Umstadt, Germany) and an API 4000 quadrupole instrument (Applied Biosystems, Foster City, USA) were used as described before [22,23]. The identification of modifications was carried out in the scheduled multiple-reaction monitoring (sMRM) mode. A modification had to satisfy the following premises to be accepted as unambiguously identified: identified to an NMR and HR-MS-controlled standard [24,25,26] of the exact modification in retention time, mass to charge ratio, formation of specific fragments at distinct fragmentation parameters as well as relative intensities of single fragments towards each other.

## 3. Results

### 3.1. Differentially Expressed RNAs of Control vs. BCE Treated EA.hy926 Cells

#### 3.1.1. Microarray Analysis

**Figure 1:**
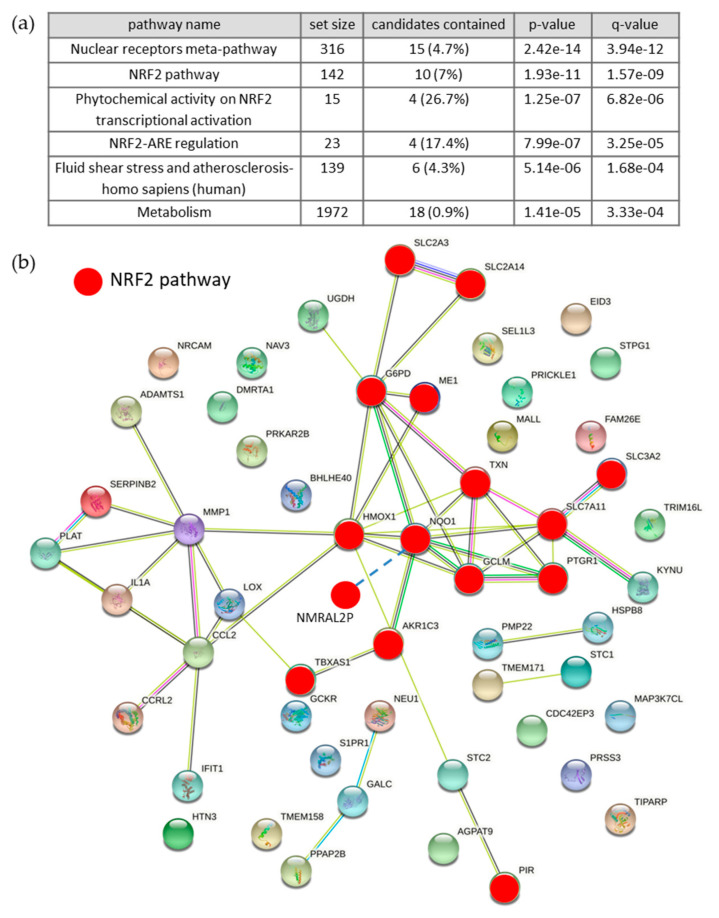
Analyses of up-regulated RNAs: (**a**) Functional annotation analyses of up-regulated genes for the identification of enriched pathways (cpdb.molgen.mpg.de; release 34; [20]). (**b**) Network analysis of up-regulated RNAs. Interaction map of the NRF2-target genes within the network are highlighted in red. The network was built utilizing the Gene String online tool (https://string-db.org; STRING 11.0b; 26 November 2020 [21]).

To understand the cellular effects of natural extracts enriched in AGEs, a water soluble fraction of bread crust (BCE) was used to stimulate human EA.hy926 endothelial cells for 24 h. Thereafter, RNA was isolated (Supplemental Figure S1A,B) and gene expression was analyzed by Affymetrix array technology. RNAs differentially expressed between the control and BCE group (fold change > 2 and *p* value < 0.01) were investigated by volcano plot (Supplemental Figure S2A) and scatter plot analyses (Supplemental Figure S2B). Further, 476 differentially expressed RNAs, 160 up-regulated and 316 down-regulated, could be identified consistently in three replicates (Supplemental Figure S2C). We further concentrated on up-regulated coding RNAs and did functional annotation analyses for the identification of enriched pathways (Figure 1a; [20]). The up-regulated genes are particularly associated with the NRF2 pathway as confirmed by pathway analysis. Next, we examined the interplay of regulated RNAs by network analyses (Figure 1b; [21]). It could be corroborated that many genes induced by BCE treatment are regulated by NRF2 transcription factor (marked in red). Notably, the transport proteins, solute carrier family 3, member 2 and solute carrier family 7, member 11 (SLC3A2 and SLC7A11) are known as ferroptosis-modulating genes and build an antiporter system [27]. The hierarchical clustering was utilized as quality control for consistent expression patterns throughout the individual samples (Supplemental Figure S3).

The analysis of microRNA-precursors regulated by BCE revealed a second layer of NRF2-signaling regulation (see Supplemental Tables S1 and S2). For all five mature microRNAs (miRs): miR-4675, miR-6754-3p, miR-6754-5p, miR-301a-3p and miR301a-5p, putative target genes could be found significantly regulated in the array data (see Supplemental Table S3). Furthermore, analysis of the target data sets for enrichment in data of transcription factor perturbation and following expression revealed a strong enrichment of the genes in NRF2-regulated expression (see Supplemental Figure S4). This aids the assumption of the regulation of the found miRNAs by NRF2.

Finally, up-regulated NRF2 target genes in EA.hy926 cells following BCE treatment are summarized in Supplemental Table S4.

#### 3.1.2. Effects of BCE on mRNA Expression Levels in EA.hy926 and HeLa Cells

**Figure 2:**
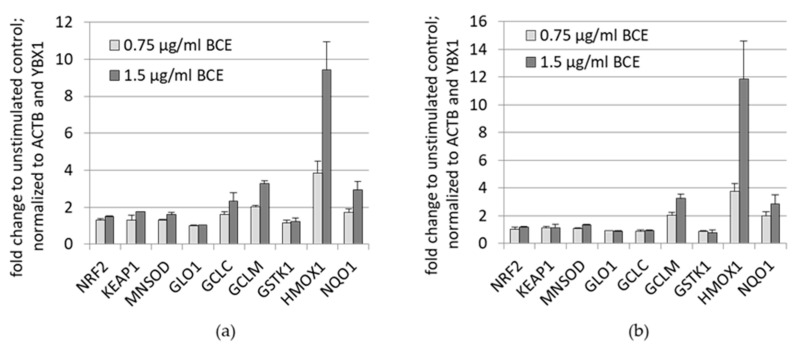
BCE increases antioxidant gene expression in HeLa and EA.hy926 cells. (**a**) HeLa, and (**b**) EA.hy926 cells were incubated with 0.75 µg/mL and 1.5 µg/mL BCE for 24 h. MRNA abundance of the indicated antioxidant genes was measured by quantitative real-time PCR (qRT-PCR) and normalized to reference genes (ACTB, YBX1). Data were analyzed from three independent experiments and depicted as means with standard deviation.

In order to validate the array data, typical NRF2 downstream targets, involved in the anti-oxidative stress response were analyzed by qRT-PCR after 24 h BCE treatment of HeLa (Figure 2a) and EA.hy926 cells (Figure 2b). Corresponding to the array data, genes encoding for anti-oxidative enzymes such as HMOX1, GCLM and NQO1 were up-regulated upon BCE treatment in a dosage dependent manner in both cell lines. Expression of NRF2 itself and its inhibitor KEAP1 were not changed upon BCE treatment.

### 3.2. Effects of BCE on Protein Expression Levels in EA.hy926 and HeLa Cells

**Figure 3:**
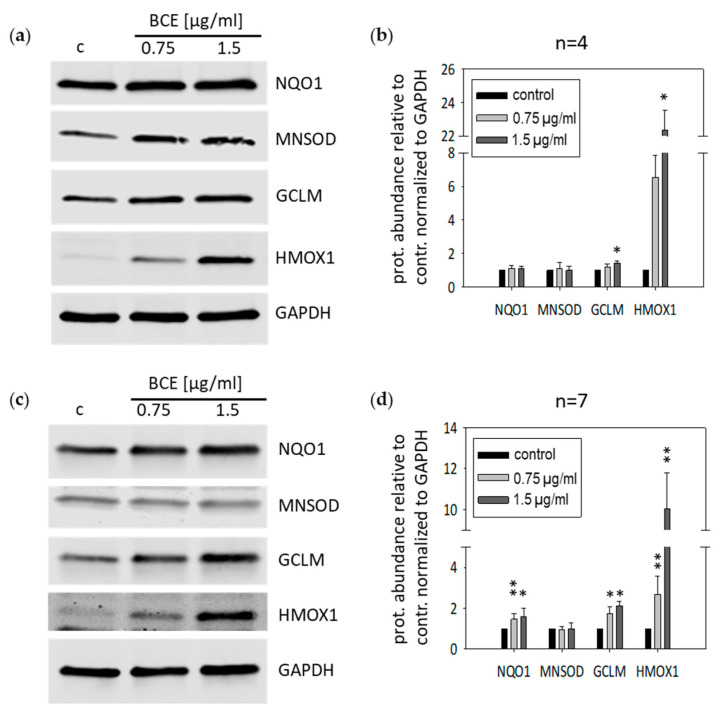
BCE affects protein expression of HMOX1, GCLM and NQO1 in HeLa and EA.hy926 cells. (**a**) HeLa, and (**c**) EA.hy926 cells were incubated without and with 0.75 µg/mL and 1.5 µg/mL BCE for 24 h. Expression of the indicated proteins upon BCE treatment was determined by Western blotting. One representative Western blot of four independent experiments with HeLa cells (**a**), and one representative Western blot of seven independent experiments with EA.hy926 cells (**c**) are shown. (**b**,**d**) Protein abundance was normalized to GAPDH and quantified relative to a control. The data were presented as mean values with standard deviation from four independent experiments with HeLa cells (**b**), and seven independent experiments with EA.hy926 cells (d). * *p* < 0.05; ** *p* < 0.01; (*t*-test) BCE vs. control sample.

To investigate the capability of BCE to induce anti-oxidative enzyme production, we did Western blot analyses with specific antibodies against HMOX1, GCLM, NQO1 and MNSOD (Figure 3). Consistent with the gene expression analyses and qRT-PCR measurements, the production of HMOX1 and GCLM was induced upon BCE treatment in HeLa (Figure 3a,b) as well as in EA.hy926 cells (Figure 3c,d) in a dosage dependent manner compared to untreated control cells. Furthermore, NQO1 protein was increased upon BCE treatment in EA.hy926 cells (Figure 3c,d).

### 3.3. Effects of BCE on NRF2 Induced Gene Expression in EA.hy926 Reporter Cells

**Figure 4:**
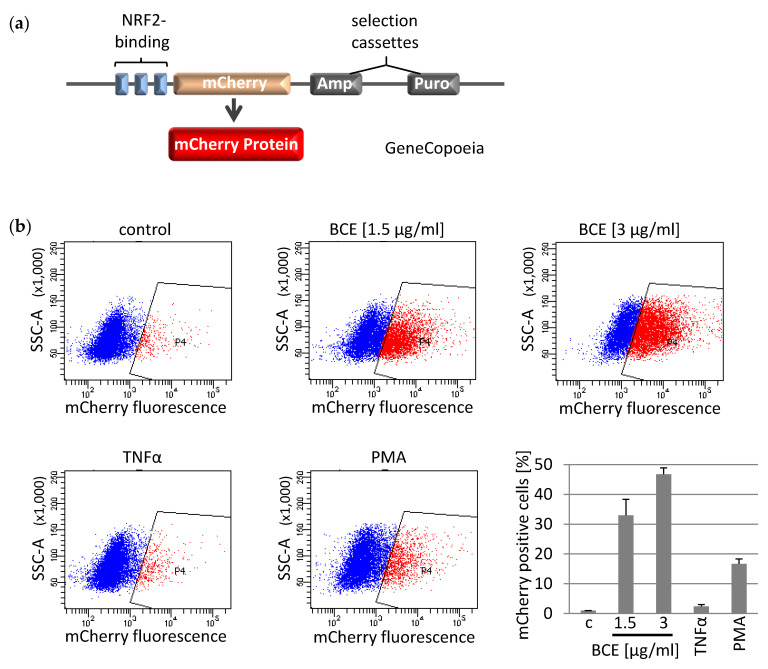
Bread crust extract (BCE) induces the transcription factor NRF2 in EA.hy926 cells. (**a**) Schematic of the reporter plasmid transfected in EA.hy926 cells. The NRF2 binding sites, antioxidant response elements (ARE) are shown as blue rectangles. The activated fluorescence reporter gene, mCherry is highlighted in orange and its protein product is depicted in red. Selection cassettes (puromycin and ampicillin) are illustrated as grey boxes. (**b**) Induction of mCherry protein expression in transfected EA.hy926 cells evaluated by flow cytometry. The scatter plot graph indicates the cell scatter on the *y*-axis vs. the PE-Texas Red-A filter (mCherry fluorescence) on the *x*-axis measured on a BD Fortessa device and using the Diva software (BD-biosciences). As indicated, untreated reporter cells (control), cells treated with BCE (1.5 µg/mL; 3 µg/mL), cells treated with TNFα (15 ng/mL) or PMA (20 ng/mL) for 24h are shown. The percentage of mCherry positive cells was determined in three independent experiments.

To further explore NRF2-dependence in BCE-mediated stimulation of antioxidant gene expression, we developed a NRF2 reporter system in EA.hy926 cells (Figure 4a). Our construct encodes a mCherry fluorescent reporter under transcriptional control of three antioxidant response elements (AREs). First, we confirmed assay performance by application of the chemical NRF2 activator phorbol myristate acetate (PMA). Treatment of reporter cells with BCE resulted in an increased production of mCherry reporter protein in a dosage dependent manner as measured by flow cytometry (Figure 4b). The fluorescence clearly indicates binding of NRF2 and thus, confirmed NRF2-dependence of the observed RNA- and protein regulation.

### 3.4. Effects of BCE on H_2_O_2_-Induced Cell Death in EA.hy926 Cells

**Figure 5:**
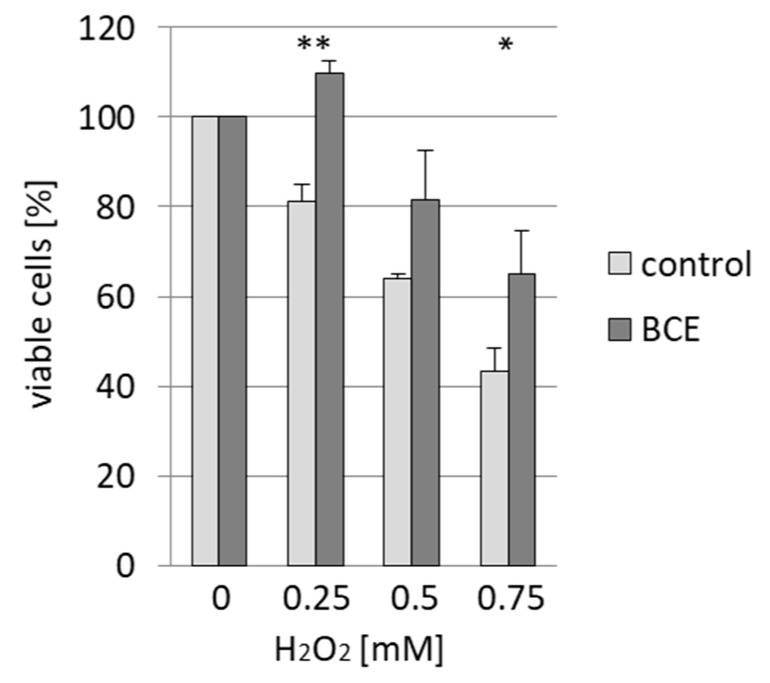
BCE pretreatment promotes resistance against oxidative stress in EA.hy926 cells. EA.hy926 cells were incubated first without or with 3 µg/mL BCE (18 h) and then without or with H_2_O_2_ (0.25 mM; 0.5 mM and 0.75 mM; 4 h). The number of viable cells was determined relative to a control (the number of cells without H_2_O_2_ treatment was set to 100%). The data were presented as mean values with standard deviation from three independent experiments. * *p* < 0.05; ** *p* < 0.01 (*t*-test) BCE treated vs. control sample.

To determine whether BCE treatment ameliorates the effect of oxidative stress on EA.hy926 cells, cells were first incubated with or without BCE and thereafter treated with H_2_O_2_. We observed that BCE pretreated cells had a survival-advantage compared to control cells (Figure 5). This result suggests a BCE-mediated increased cellular resistance against oxidative stress, which corresponds to elevated antioxidant gene expression modulated by NRF2.

### 3.5. Inhibition and Induction of NRF2-Pathway

**Figure 6:**
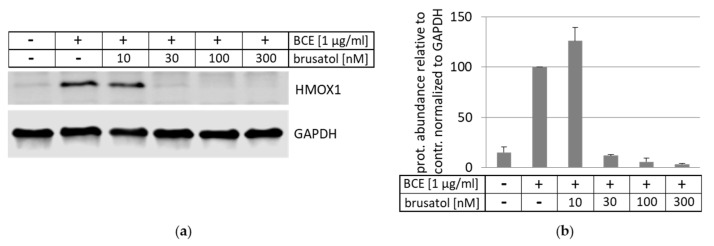
Effect of NRF2 inhibitor brusatol on BCE-induced HMOX1 expression. (**a**) HeLa cells were treated without or with BCE (1 µg/mL; 24 h; indicated by -/+) in the absence or presence of the NRF2 inhibitor brusatol (10 nM, 30 nM, 100 nM, 300 nM; 24 h). HMOX1 expression was examined by Western blot analysis and normalization to GAPDH protein. One representative Western blot of three independent experiments with HeLa cells is shown. (**b**) HMOX1 protein abundance was quantified relative to a control (BCE-treated cells in absence of brusatol were set to 100%) and normalized to GAPDH. The data were presented as mean values with standard deviation from three independent experiments.

Next, we examined whether the BCE-mediated activation of the antioxidant gene HMOX1 was regulated by the NRF2 signaling pathway. Therefore, HeLa cells were treated with BCE in the absence and presence of increasing amounts of the NRF2 inhibitor brusatol (Figure 6). Indeed, upon application of brusatol, the effect of BCE on HMOX1 protein expression was abrogated, indicating the requirement of NRF2 for BCE-induced antioxidant gene expression.

**Figure 7:**
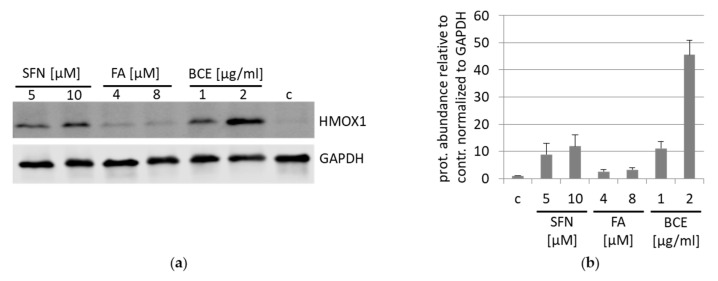
Induction of HMOX1 expression by different NRF2 inducers compared to BCE. (**a**) HeLa cells were treated with NRF2 inducer sulforaphane (5 µM, 10 µM SFN; 24 h), falcarinol (4 µM, 8 µM FA; 24 h) and with BCE (1 µg/mL, 2 µg/mL; 24 h) and compared to untreated cells. HMOX1 expression was examined by Western blot and normalized to GAPDH protein. One representative Western blot of four independent experiments with HeLa cells is shown. (**b**) HMOX protein abundance was quantified relative to a control and normalized to GAPDH. The data were presented as mean values with standard deviation from four independent experiments.

The NRF2 pathway can be induced by sulforaphane (SFN) a compound identified in broccoli and falcarinol (FA) present in carrots. We applied those substances and BCE to compare NRF2 induction as indicated by HMOX1 expression in HeLa cells (Figure 7). SFN and BCE exhibit a similar capacity to induce HMOX1, whereas FA stimulated HMOX1 expression to a lesser extent.

### 3.6. Effects of BCE on Signaling Pathways

**Figure 8:**
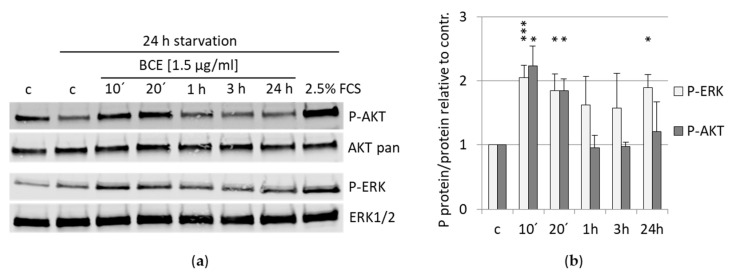
BCE induces phosphorylation of AKT and ERK kinase in EA.hy926 cells. (**a**) EA.hy926 cells were starved for 24 h (reduction of serum concentration in the cell culture medium in order to synchronize the cells) or maintained in normal medium followed by BCE treatment for indicated times. Cells incubated with 2.5% FCS for 20 min served as an induction control. Phosphorylation of AKTs at Serine 473 and phosphorylation of ERK1/2 at Threonine 202/Tyrosine 204 was determined by normalization to total AKT protein or total ERK1/2 protein, respectively. One representative Western blot of four independent experiments with EA.hy926 cells is shown. (**b**) Quantification of phosphorylated proteins vs. total non-phosphorylated proteins relative to control (under starvation). The data were presented as mean values with standard deviation from four independent experiments. * *p* < 0.05; *** *p* < 0.001 (*t*-test) BCE-treated vs. control sample.

Moreover, a recent publication revealed that induction of NRF2 by sulforaphane (SFN) exerts antioxidant stress through PI3K/AKT signaling [28]. To analyze whether BCE affects different signaling pathways, we investigated the phosphorylation status of AKT at Serine 473 and ERK1/2 at Threonine 202 and Tyrosine 204 upon BCE treatment by Western blotting (Figure 8). Phosphorylation of AKT was already significantly increased after 10 and 20 min of BCE application. Notably, after 1 h of BCE treatment, phosphorylation of AKT was comparable to untreated cells. Similarly, phosphorylation of ERK was also significantly induced after 10 and 20 min; however, in comparison to AKT phosphorylation, ERK phosphorylation stayed constant over time even after 24 h BCE treatment (Figure 8a,b). Thus, BCE potentially activates NRF2 by stimulating PI3K/AKT and ERK signaling.

### 3.7. Identification of AGE-Modifications in BCE by Mass Spectrometry

**Figure 9:**
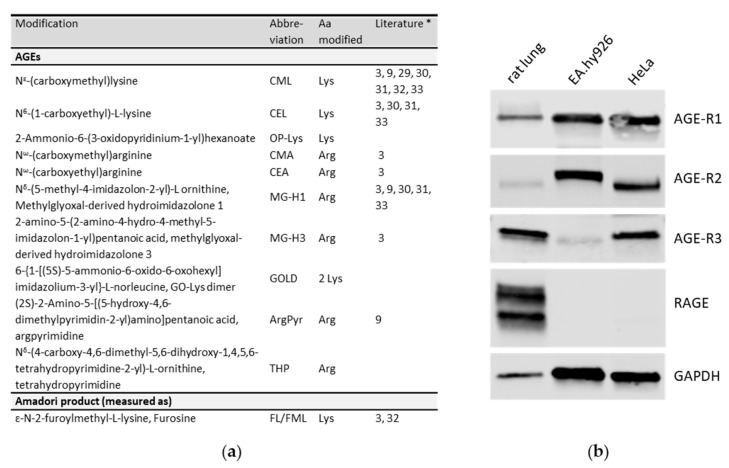
Identification of AGE-modifications in BCE: (**a**) Putatively active protein-modifications in BCE hydrolysate identified by HPLC-MS/MS (Literature*: AGEs and Amadori products already described in bread). (**b**) Expression of different receptors for AGEs was determined by Western blotting with indicated antibodies in EA.hy926 and HeLa cells. Lung lysate from rats was used as a positive control for RAGE expression and GAPDH served as a loading control.

As BCE was used as a typical natural extract containing high amounts of AGEs, we examined AGE-modifications present in BCE by acidic hydrolysis LC–MS analysis and found a variety of modified lysines and arginines (summarized in Figure 9a). The AGE-modifications, CML, CEL, CMA, CEA, MG-H1, MG-H3 and ArgPyr, were consistently identified [3,9,29,30,31,32,33] and some new AGE-compounds were discovered. AGE-related effects are transduced by the receptor for advanced glycation end products (RAGE) and other AGE-receptors such as AGE-R1-3. To test whether these receptors are expressed in EA.hy926 and HeLa cells, we performed Western blot analyses (Figure 9b). As a positive control for RAGE expression, we used a lysate from rat lungs. EA.hy926 cells express AGE-R1, AGE-R2 but only very faint expression was detected for AGE-R3, whereas HeLa cells express all three AGE-receptors. RAGE and its glycosylated form were only visible in lung lysate but not in EA.hy926 and HeLa cells. Hence, it is possible that the induction of NRF2 can be mediated by AGE-modifications present in BCE via one or more receptors for AGEs in EA.hy926 and HeLa cells whereas it cannot be excluded that additional receptors are involved.

## 4. Discussion

**Figure 10:**
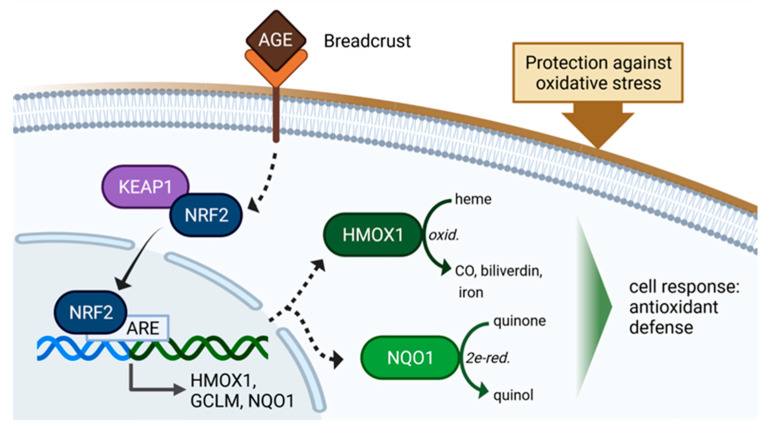
Model for the molecular mode of BCE action. The figure was created with BioRender.com.

The present study investigated antioxidant responses in a human endothelial cell line (EA.hy926) and in a cancer cell line (HeLa). It examined the role of BCE in the activation of the antioxidant transcription factor NRF2. Induction of NRF2 leads to the expression of detoxifying and antioxidant proteins [34]. It thereby serves as a therapeutic target in combating diseases such as Parkinson’s [35], type 2 diabetes mellitus [36], intestinal inflammation [37] or cardiovascular diseases [38]. In previous studies, induction of HMOX1 in endothelial cells by AGE-BSA was shown [11], and NRF2 activation in macrophages, Caco-2 cells, and intact human gut tissue by MRPs and coffee was demonstrated [10]. Among BCE mediated NRF2 induction, we could clearly exhibit an up-regulation of HMOX1 (Figure 10), a well-known antioxidant that catalyzes heme oxidation to carbon monoxide, bilirubin and iron. In cardiomyocytes, HMOX1 overexpression protects against ischemia and reperfusion injury [39]. Furthermore, Perrella and Yet revealed that mice with cardiac-specific HMOX1 overexpression have an improvement in cardiac function and reduced inflammatory and oxidative damage after coronary artery ligation and reperfusion [40]. Besides its cardioprotective function, HMOX1 was also demonstrated to have an antitumorigenic role, as shown by Marelli [41] in gut macrophages where HMOX1 expression protects against colitis-associated cancer. Moreover, in a breast cancer cell line, HMOX1 inhibits TGF-β-induced endothelial-mesenchymal transition [42].

The antioxidant flavoprotein NQO1 (Figure 10), which was also induced upon BCE treatment in the present study, catalyzes the reduction of substrates such as quinones and nitro-compounds [43]. NQO1 exerts antioxidant functions by the production of antioxidant forms of ubiquinone [44] and vitamin E [45]. Furthermore, anti-tumorigenic characteristics are known for NQO1. On the one hand, it stabilizes tumor suppressor p53 by inhibiting proteasomal degradation [46]. On the other hand, NQO1 induction is used in cancer therapy because bio-reductive anticancer drugs (e.g., mitomycin C) are activated by NQO1 [47,48].

In 2002, Lindenmeier and colleagues, found that pronyl-lysine from an ethanolic bread crust extract prevents oxidative damage by acting on phase 1 and phase 2 enzymes [4]. Although we could not identify this compound in our study, likely due to the alternative extraction method used for the BCE fraction, some new AGE-compounds such as OP-Lys, GOLD and THP were identified in BCE (listed in Figure 9a). AGEs can bind a variety of receptors such as RAGE, scavenger receptors, or AGE receptors 1–3, which differ in various cell types (reviewed in [49,50]). We demonstrated expression of AGE receptor 1 and 2, but only a faint amount of AGE receptor 3 in EA.hy926 cells and AGE receptor 1, 2 and 3 in HeLa cells. However, RAGE expression was not detectable in both cell lines compared to the lung lysate positive control. AGE–RAGE interaction can induce oxidative stress and inflammatory response [51]. In contrast, AGE receptor 1 is known as a negative regulator of inflammatory response [52] and suppresses cell oxidant stress [53]. AGE receptor 2 functions as an AGE binding receptor [54], which can be phosphorylated and thereby plays a role in intracellular receptor signaling [55,56] and vesicle trafficking [57]. AGE receptor 3, also known as galectin-3 (Gal-3) can bind AGEs with high affinity [58]. Furthermore, it was indicated that Gal-3 promotes ROS-production, for example, in neutrophils [59] and monocytes [60].

Notably, among the top up-regulated genes identified by microarray analyses, a set of ferroptosis-modulating genes was uncovered. Ferroptosis plays a role in the development of several diseases such as acute kidney injury (AKI), acute lung injury (ALI), neurological diseases, tumors and heart diseases [61,62,63,64,65,66]. Several studies illustrated that NRF2 can inhibit or mitigate the ferroptotic cascade [64,65,67]. Ferroptosis is induced by either inhibition of glutathione peroxidase 4 (GPX4) or the cystine/glutamate transporter system (xC-/xCT) [68,69]. This antiporter system is composed of SLC7A11 and SLC3A2 [27]. It transports glutamate to extracellular space and cystine into the cell [70], which in turn is essential for GSH synthesis. GSH enables the function of GPX4 and is synthesized by glutamate–cysteine ligase, consisting of catalytic (GCLC) and regulatory subunit (GCLM). Several studies showed that HMOX1 also acts against ferroptosis by catalyzing the oxidation of heme to biliverdin [71,72] and by regulation of the iron metabolism [73]. Additionally, studies on liver revealed that NRF2 activation by glycyrrhizin mitigates ferroptosis by HMOX1 and GPX4 up-regulation [74]. Furthermore, in hepatocellular carcinoma cells, knockdown of p62, NQO1, FTH1 and HMOX1 promoted ferroptosis in response to erastin and sorafenib [65].

Li and colleagues found that decreased ferroptosis in folic acid-induced kidney injury is achieved via AKT/GSK-3-β-mediated NRF2 activation [75]. Consistently, we demonstrated an activation of AKT upon BCE treatment in EA.hy926 cells. Hence, the observed induction of NRF2 by BCE could be due to the fact that phosphorylated AKT/GSK-3β inhibits the nuclear export of NRF2 [76]. Furthermore, activated AKT phosphorylates and inhibits GSK-3β, which in turn results in an inhibition of β-TrCP-dependent NRF2 degradation [77,78,79]. NRF2 activation by promotion of PI3K/AKT signaling was also described with NRF2 agonists such as sulforaphane [28].

Chen and Maltagliati described NRF2 as the “golden goose” in prevention toward cardiac injury [77]. Indeed, NRF2 induction was shown to suppress atherosclerotic signaling, causes a 20% reduction in infarct size in reperfusion and ameliorates cardiac hypertrophy and fibrosis [80,81,82].

Consequently, the results of the present study suggest that BCE treatment can potentially interfere with oxidative stress and ferroptosis by activating NRF2 and downstream proteins such as HMOX1, GCLM, NQO1, SLC7A11 and SLC3A2. Furthermore, BCE could serve as a promising and molecularly well-described functional food to induce the antioxidant defense. Whether BCE has the potential to protect against tissue injury remains to be further investigated to provide new strategies to prevent or mitigate acute organ injury.

## Data Availability

The data presented in this study are available in the Supplementary Material. Raw microarray data are available on reasonable request from the corresponding author.

## Supplementary Materials

The following are available online at https://www.mdpi.com/article/10.3390/nu13113874/s1: Figure S1: RNA template examination for microarray analyses. Figure S2: Microarray data. Figure S3: Hierarchical clustering analyses of up-regulated RNAs. Table S1: Array data—up-regulated precursor microRNAs. Table S2: Array data—down-regulated precursor microRNAs. Table S3: Regulated microRNA-precursors following BCE-treatment. Figure S4: Enrichment analysis of miR-targets in data sets of gene expression following TF Perturbation. Table S4: BCE treatment of EA.hy926 cells induces several NRF2 target genes.

## Abbreviations

ACTB	actin beta
AGEs	advanced glycation end products
AGE-R1-3	AGE receptors 1-3
AKT	AKT serine/threonine kinase
AVG	average
ARE	antioxidant response element
Arg	arginine
BCE	bread crust extract
Caco	colorectal adenocarcinoma (human)
DME’s	drug-metabolizing enzymes
EA.hy926	immortalized human vascular endothelial cells
EMT	epithelial-mesenchymal transition
ERK	mitogen-activated protein kinase
FA	falcarinol
FCS	fetal calf serum
GAPDH	glyceraldehyde-3-phosphate dehydrogenase
GCLC	glutamate-cysteine ligase catalytic subunit
GCLM	glutamate-cysteine ligase modifier subunit
GLO1	glyoxalase I
GSH	glutathione
GSTK1	glutathione S-transferase kappa 1
H2O2	hydrogen peroxide
HMOX1	heme oxygenase 1
KEAP1	kelch-like ECH associated protein 1
Lys	lysine
MNSOD	superoxide dismutase 2
MRPs	Maillard reaction products
NADPH	nicotinamide adenine dinucleotide phosphate
NQO1	NAD(P)H quinone dehydrogenase 1
NRF2	nuclear factor E2-related factor 2
NF-κB	nuclear factor kappa B
P	phosphorylation
PBS	phosphate-buffered saline
PE	phycoerythrin
PI3K	phosphoinositide 3-kinase
PMA	phorbol 12-myristate 13-acetate
qRT-PCR	quantitative Real time-PCR
RAGE	advanced glycosylation end product receptor
RIN	RNA integrity number
ROS	reactive oxygen species
SDS-PAGE	sodium dodecyl sulfate polyacrylamide gel electrophoresis
SFN	sulforaphane
TNFα	tumor necrosis factor a
Trp	tryptophan
Tyr	tyrosine
YBX1	Y-box binding protein 1

## Appendix A

**Table A1:** Antibodies used for detection of specific proteins.

Primary antibodies			
antibody	Company (order number)	dilution	isotype
GCLM	Abcam (ab126704)	1:1500	rabbit monoclonal IgG
HMOX1	Abcam (ab68477)	1:2500	rabbit monoclonal
HMOX1	Enzo Life Sciences (ADI-SPA-896)	1:1000	rabbit polyclonal
NQO1	Santa Cruz (sc-32793)	1:500	mouse monoclonal
GAPDH	Cell Signaling (#5174)	1:1000	rabbit monoclonal IgG
GAPDH	Sigma-Aldrich (G8795)	1:20000	mouse monoclonal IgM
MNSOD	Biomol (600-401-G13)	1:1000	rabbit
Phospho-AKT (Ser473)	Cell Signaling (#4060)	1:2000	rabbit polyclonal IgG
AKT-pan	Cell Signaling (#2920)	1:2000	mouse monoclonal IgG1
Phospho-ERK1/2 (Thr202/Tyr204)	Cell Signaling (#4370)	1:2000	rabbit monoclonal IgG
ERK1/2	Abcam (ab184699)	1:10000	rabbit monoclonal
RAGE	Abcam (ab65965)	1:3000	rabbit polyclonal
AGE-R1	Santa Cruz (sc-74408)	1:1000	mouse monoclonal IgG1
AGE-R2	Abcam (ab134071)	1:2000	rabbit monoclonal IgG
AGE-R3	Santa Cruz (sc-19283)	1:1000	goat polyclonal IgG
Secondary antibodies			
antibody	Company (order number)	dilution	isotype
IRDye 800CW	Li-Cor (926-32211)	1:15000	goat anti-rabbit
IRDye 680LT	Li-Cor (926-68020)	1:15000	goat anti-mouse

**Table A2:** List of the qRT-PCR primers.

Genes	Forward Primers (5′–3′)	Reverse Primers (5′–3′)	Source
NRF2	5′-CAGCGACGGAAAGAGTATGA-3′	5′-TGGGCAACCTGGGAGTAG-3′	[83]
KEAP1	5′-GGCTGTCCTCAATCGTCTCC-3′	5′-TCTGTTTCCACATCGTAGCG-3	[83]
HMOX1	5′-CAACATCCAGCTCTTTGAGG-3′	5′-GGCAGAATCTTGCACTTTG-3′	[83]
ACTB	5′ -CTGGAACGGTGAAGGTGACA-3′	5′-AAGGGACTTCCTGTAACAATGCA-3′	[83]
MNSOD	5′-CACCGAGGAGAAGTACCAGG-3′	5′-TAGGGCTGAGGTTTGTCCAG-3′	[84]
GCLC	5′-GTCCTTTCCCCCTTCTCTTG-3′	5′-AGGACGTTCTCAAGTGGGG-3′	[84]
GSTK1	5′-TGGTCTCCTTGAGCTGGTTC-3′	5′-AATGAAGACATCACCGAGCC-3′	[84]
GLO1	5′-TGGATTAGCGTCATTCCAAG-3′	5′-CAGTTGCTGCTCCGACG-3′	[84]
YBX1	5′-ACTGCGAAGGTACTTCCTGG-3′	5′-TGGTTCAATGTAAGGAACGGA-3′	[84]
GCLM	5′-ACTCGTGCGCTTGAATGTC-3′	5′-CTGTGTGATGCCACCAGATT-3′	[84]
NQO1	5′-GCATAGAGGTCCGACTCCAC-3′	5′-GGACTGCACCAGAGCCAT-3′	[84]
