# Supplementary material for: AGE-Rich Bread Crust Extract Boosts Oxidative Stress Interception via Stimulation of the NRF2 Pathway
Source: Nutrients. 2021 Oct 29;13(11):3874. doi: 10.3390/nu13113874 (PMC8622267; doi:10.3390/nu13113874)
Supplement: Supplementary file 1 [file nutrients-13-03874-s001.zip › nutrients-1439028-supplementary.pdf]

Supplemental data

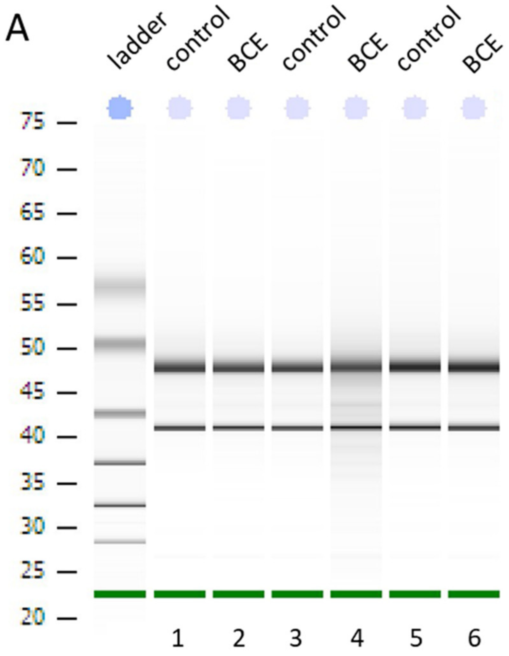

B

sample	RIN value
1	10
2	9,9
3	9,9
4	9
5	10
6	10

*RIN…RNA integrity number

Figure S1: RNA template examination for microarray analyses. Integrity of six RNA samples of control vs. bread crust extract (BCE) treated EA.hy926 cells, was verified by bioanalyzer (A). The RNA integrity number (RIN) values are summarized in table (B).

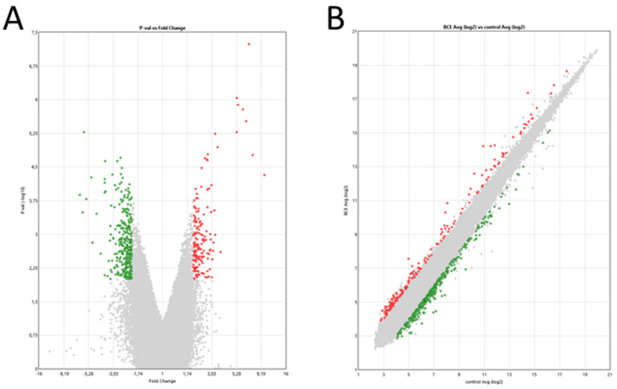

C

Group	Total	Up-Regulated	Down-Regulated
NonCoding	66845	59	120
Multiple Complex	29510	55	49
Coding	18858	21	49
Unassigned	10001	15	90
Pseudogene	4340	5	2
Precursor microRNA	3297	4	5
Small RNA	2386	1	1
Ribosomal	507	0	0
tRNA	6	0	0

Figure S2: Microarray data: Differentially expressed RNAs of control vs. bread crust extract (BCE) treated EA.hy926 cells. Cluster analyses of differentially expressed RNAs. (A) Volcano plot analysis of control vs. BCE group. The x-axis represents the difference between the two stimulation conditions (fold change) and the y-axis represents the -log10 p-value. (B) Scatter plot analysis of BCE average (log2) vs. control average (log2) group. Significantly up-regulated RNAs are represented as red dots and down-regulated RNAs are represented as green dots. (C) Summary table of the number of differentially expressed RNAs.

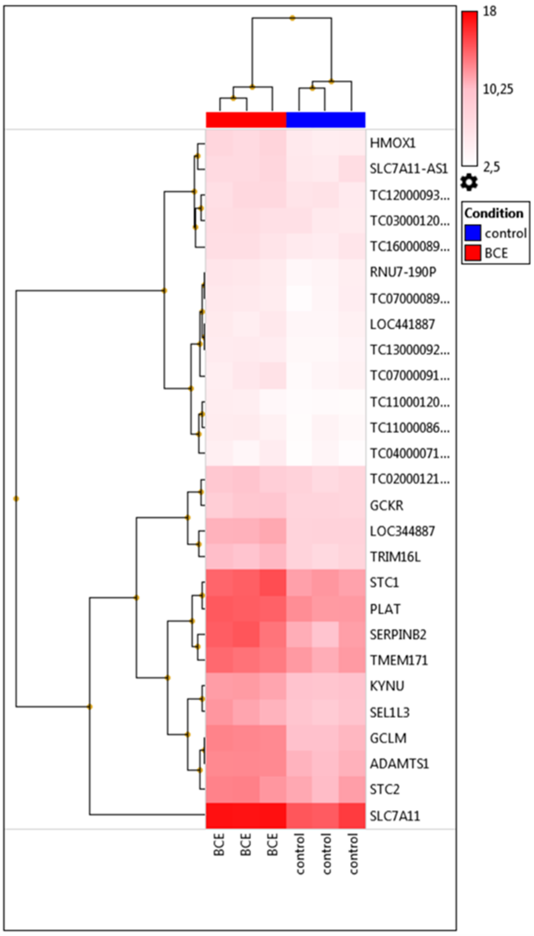

Figure S3: Hierarchical clustering analyses of up-regulated RNAs.

Table S1: Array data - up-regulated precursor microRNAs.

ID	BCE Avg (log2)	control Avg (log2)	Fold Change	P-val	FDR P-val	Gene Symbol	Description	Group
TC1200011336.hg.1	6,28	5	2,42	0,0026	0,6402	AC068797.1		Precursor_microRNA
TC1000006952.hg.1	5,03	3,95	2,12	0,0084	0,7304	MIR4675	microRNA 4675	Precursor_microRNA
TC0200015294.hg.1	4,53	3,45	2,12	0,0005	0,5599	AC073973.1		Precursor_microRNA
TC1100008307.hg.1	7,78	6,78	2,01	0,0055	0,7028	MIR6754	microRNA 6754	Precursor_microRNA

Table S2: Array data - down-regulated precursor microRNAs.

ID	BCE Avg (log2)	control Avg (log2)	Fold Change	P-val	FDR P-val	Gene Symbol	Description	Group
TC1200010743.hg.1	8,68	10,23	-2,93	0,0057	0,7028	AC021066.1		Precursor_microRNA
TC1700011284.hg.1	3,53	4,81	-2,42	0,007	0,7234	MIR301A	microRNA 301a	Precursor_microRNA
TC2200008882.hg.1	4,64	5,8	-2,24	0,0003	0,4964	Z93241.1		Precursor_microRNA
TC1100007575.hg.1	4,95	6,01	-2,09	0,0093	0,7304	AC110283.1		Precursor_microRNA

TC0300011830.hg.1	7,41	8,41	-2,01	0,0001	0,3942	AC055723.1		Precursor_microRNA

miRNA precursor (reg)<br>mature miR	Significantly regulated<br>miR-targets (reg)	additional targets<br>connected to NRF2
MIR4675 (↑)		
hsa-miR-4675	MYLK2 (↓)	RARA, NCOR1
MIR6754 (↑)		
hsa-miR-6754-3p	PEG10(↓), GALNT10(↓), SLC3A2(↑)	**CUL3**, FOX03, HIF1A
hsa-miR-6754-5p	DHRS2(↓), **KRT80(↓),** C20orf96(↓), **PLXNA4(↓),** CFAP57(↓) PRICKLE1(↑)	CREB3, VEGFA, GPX3, MAPK3, SQSTM1, GSK3B
MIR301A(↓)		
hsa-miR-301a-3p	ITGB8(↓), FSTL5(↓), TENM1(↓) SEL1L3(↑), BHLHE40(↑), STC1(↑)	EIF2AK3, MAF, PPAGR, **CUL3**
hsa-miR-301a-5p	**KRT80(↓), PLXNA4(↓)** SLC7A11(↑)	EIF2AK1, **CUL3**

Table S3: Regulated microRNA-precursors following BCE-treatment. Mature miRs of regulated precursor miRNAs were analyzed for their target proteins using miRDB (1, 2). Targets, which were found to be significantly regulated in the presented array and targets that showed a direct connection to NRF2 in STRING DB-analysis are presented. Targets that are predicted for more than one miR are emphasized in bold. (↑): upregulation, (↓) downregulation.

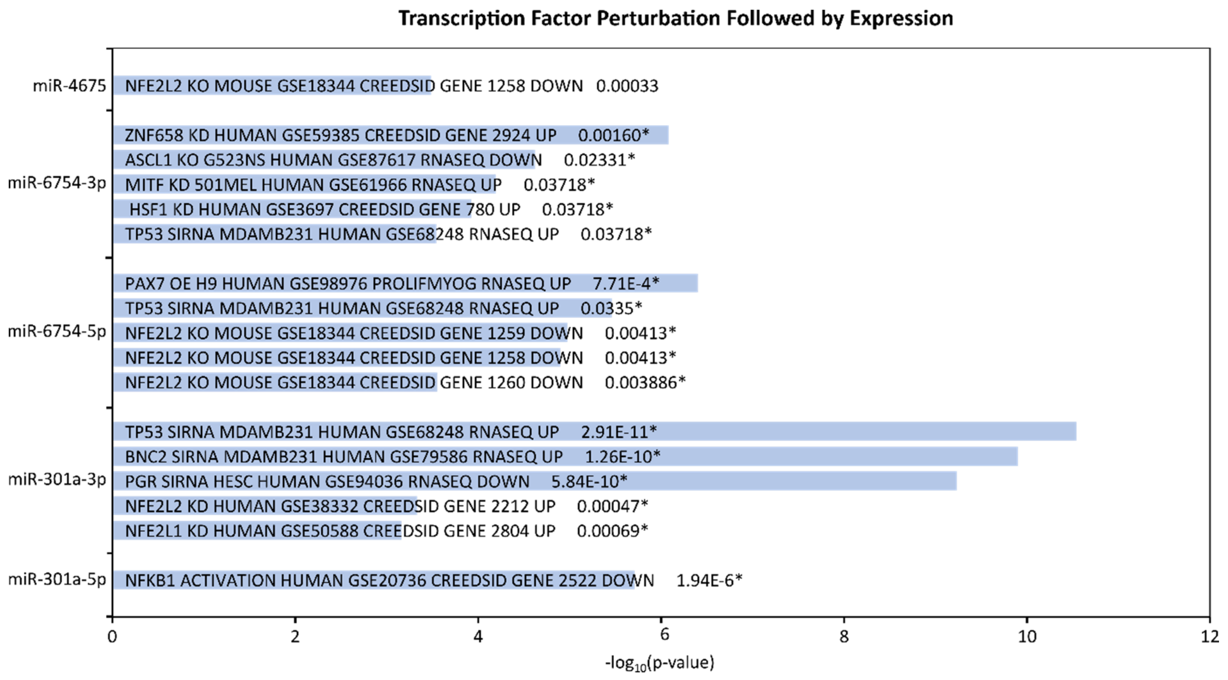

Figure S4: Enrichment analysis of miR-targets in data sets of gene expression following TF Perturbation. Interestingly, 3 of 5 target-gene sets analyzed are enriched in expression data-sets following NRF2 perturbation. Data set names and non-adjusted p-values are indicated for a maximum of 5 data sets per mature miR. Analysis was carried out by enrichr. *: Benjamini-Hochberg-adjusted p-value<0.05

Gene	Description	GenBank accession number	Fold Change	*P* value
		**antioxidant**		
NMRAL2P	NmrA-like redox sensor 2 pseudogene	NR_033752	6.96	5.81E-08
HMOX1	heme oxygenase 1	NM_002133	6.05	1.65E-06
SLC7A11	solute carrier family 7, member 11	NM_014331	7.59	1.72E-05
GCLM	glutamate-cysteine ligase, modifier subunit	NM_001308253	5.29	9.11E-07
NQO1	NAD(P)H dehydrogenase, quinone 1	NM_000903	2.41	3.34E-05
TXN	thioredoxin	NM_001244938	2.01	0.001
		**DMEs & drug transporters**		
PTGR1	prostaglandin reductase 1	NM_001146108	2.09	9.72E-05
TBXAS1	thromboxane A synthase 1	NM_001061	2.1	0.0066
		**NADPH-generating enzymes**		
G6PD	glucose-6-phosphate dehydrogenase	NM_000402	2.17	9.00E-04
ME1	malic enzyme 1	NM_002395	2.15	0.0003
		**metal-binding proteins**		
PIR	Pirin	NM_001018109	2.07	0.001
		**transport**		
SLC2A3	solute carrier family 2, member 3	NM_006931	2.04	0.0002
SLC2A14	solute carrier family 2, member 14	NM_001286233	2.1	0.0003
SLC3A2	solute carrier family 3, member 2	NM_001012662	2.51	0.0014
		**other enzymes**		
AKR1C3	aldo-keto reductase family 1 member C3	NM_001253908	2.06	0.0009

DMEs…drug-metabolizing enzymes

Table S4: BCE treatment of EA.hy926 cells induces several NRF2 target genes. Summary of up-regulated NRF2 target genes in BCE treated EA.hy926 cells, indicated by fold change > 2 and p value < 0.01.
